# p53-Dependent Anti-Proliferative and Pro-Apoptotic Effects of a Gold(I) *N*-Heterocyclic Carbene (NHC) Complex in Colorectal Cancer Cells

**DOI:** 10.3389/fonc.2019.00438

**Published:** 2019-05-29

**Authors:** Yasamin Dabiri, Mohamed A. Abu el Maaty, Hoi Yin Chan, Jessica Wölker, Ingo Ott, Stefan Wölfl, Xinlai Cheng

**Affiliations:** ^1^Department of Pharmaceutical Biology, Institute of Pharmacy and Molecular Biotechnology, Heidelberg University, Heidelberg, Germany; ^2^School of Pharmacy, University College London, London, United Kingdom; ^3^Institute of Medicinal and Pharmaceutical Chemistry, Technische Universität Braunschweig, Braunschweig, Germany

**Keywords:** gold(I) *N*-heterocyclic carbene complex, p53, p21, p73, colorectal cancer, apoptosis

## Abstract

The tumor suppressor p53 has a diverse mutational profile in human malignancies, which is known to influence the potency of various chemotherapeutics, such as platins and anti-metabolites. However, the impact of the mutations in the *TP53* gene (coding for p53) on the anti-cancer efficacy of gold complexes remains incompletely understood. We therefore investigated the anti-tumor properties of a gold(I) *N*-heterocyclic carbene (NHC) complex–termed MC3–in human colorectal cancer (CRC) cell lines encompassing three different p53 variations: HCT116 wild-type (WT), HCT116 p53^−/−^, and HT-29 (mutant; R273H). MC3 treatment induced intracellular reactive oxygen species (ROS) levels, and p21 expression, leading to cell cycle arrest in all cell lines, regardless of their p53 status. The pro-apoptotic response, however, was found to occur in a p53-dependent manner, with WT p53 harboring cells showing the highest responsiveness. Additionally, p73, which was speculated to substitute p53 in p53-deficient cells, was found to be markedly reduced with MC3 treatment in all the cell lines and knocking down its levels did not impact MC3's anti-tumor effects in HCT116 p53^−/−^ cells. Collectively, our results suggest that this small molecule has anti-cancer properties in the context of deficient or mutant p53 and may therefore have chemotherapeutic potential for clinical application.

## Introduction

Gold complexes represent a novel anti-cancer class of drugs that was inspired by the more conventional platinum-based chemotherapeutics, such as cisplatin and its derivatives (1). Gold(I) thiolates (e.g., auranofin) are long-established in the management of rheumatoid arthritis (2). More recently, pre-clinical and clinical studies on several malignancies have revealed the potential use of this class of organometallic drugs in cancer chemotherapy, which prompted the synthesis of several active species carrying phosphine, thiolate, chloride, as well as *N*-heterocyclic carbene (NHC) ligands (1). Gold(I) NHC complexes are becoming prominent as they overcome the most challenging limitations of organometallic drugs regarding stability and biological activity under physiological conditions (1, 2). The anti-cancer properties of this class of compounds have been mainly attributed to the strong inhibition of thioredoxin reductase (TrxR), which reduces oxidized thioredoxin (Trx), thereby playing an important role in cellular redox systems that are responsible for reducing thiol (-SH) groups (1, 3, 4). Aberrant expression of TrxR has been found in different types of cancer including pancreatic, lung, and colon, which enables their survival under oxidative stress through multiple mechanisms (5).

Mutations in tumor suppressor genes, such as adenomatous polyposis coli (*APC*) and *TP53* are highly prevalent in colorectal cancer (CRC), making them attractive therapeutic targets (6). *TP53* codes for the p53 protein, is a transcription factor that is activated by a wide variety of stimuli, and initiates a complex signaling program, targeting many biological processes, such as cellular metabolism, apoptosis, proliferation, cell cycle, and redox balance (7). More than 70% of *TP53* mutations are missense mutations, which give rise to “mutant p53,” a protein that lacks wild-type (WT) activity and may possess dominant-negative effects over the remaining WT protein (8). More interestingly, mutant p53 might acquire novel tumor-promoting qualities, such as hyper-proliferation, enhanced invasion/metastasis, and chemo-resistance (8).

p53 mutations are a major determinant of anti-cancer drug efficacy (9). The potency of chemotherapeutics routinely used in the treatment of CRC, such as cisplatin (10–12), oxaliplatin (13), and 5-FU (13) is known to be strongly influenced by p53 status. However, the impact of p53 variations on the anti-tumor potential of gold complexes has been controversial. A number of studies have implicated the involvement of the p53 pathway in gold complexes-mediated apoptosis (3, 14, 15), whereas several other drugs, such as auranofin have been reported to induce apoptosis independently of p53 (16, 17).

We have previously reported that the anti-cancer effects of the gold(I) NHC complex, MC3, in pancreatic cancer cells arise from its induction of intracellular reactive oxygen species (ROS), which activates p38-signaling, leading to apoptotic cell death (18). Since p53 is a redox-sensitive tumor suppressor whose activity is altered by intracellular ROS levels (19), we were inclined to investigate the influence of p53 status on the anti-tumor effects of MC3. The human CRC cell lines HCT116 WT, HCT116 p53^−/−^, and HT-29 (mutant; R273H) were employed, which represent three different p53 variants. We observed that MC3 induces tumor cell growth predominantly in a p53-dependent manner. Pro-apoptotic signaling, including p38 activation, was found to be triggered by MC3 in both HCT116 clones, however with higher efficiency in the presence of WT p53. Mutant p53 harboring HCT116 and HT-29 cells failed to activate p38 signaling and showed significantly less cytotoxicity and apoptosis compared with WT and p53-null HCT116 cell lines. Nevertheless, ROS induction, p21 activation and cell cycle inhibition were found to occur irrespective of the p53 status. Together, our findings demonstrate the potential use of MC3 in the treatment of CRC carrying distinct p53 profiles.

## Materials and Methods

### Materials

**[**di-(1,3-diethylbenzylimidazol-2-ylidene)] gold(I) iodide (MC3) was synthesized as previously described (3, 4). The purity of the compound was confirmed by elemental analysis (maximum 0.5% deviation from the calculated values for C, H, and N). Auranofin (CAS 34031-32-8) was purchased from Santa Cruz Biotechnology (Germany). Thiazolyl blue tetrazolium bromide dye (MTT, CAS 298-93-1), reduced glutathione (GSH, CAS 70-18-8), *N*-acetyl-L-cystein (NAC, CAS 616-91-1), and MG132 (CAS 1211877-36-9) were from Sigma-Aldrich (Germany). Primary antibodies against p21^Waf1/Cip1^ (#2947), PARP (#9542), Bcl-xL (#2764), Bax (#5023), Bcl-2 (#2872), Puma (#12450), cytochrome c (#4272), COX IV (#11967), pp38 MAPK (T180/Y182, #9211), p73 (#14620) were obtained from Cell Signaling Technologies (NEB, Germany), whereas those against p53 (SC-126), β-actin (SC-47778) and vinculin (SC-73614) were from Santa Cruz Biotechnology. Anti- mouse and -rabbit IgG horseradish peroxidase (HRP)-linked secondary antibodies were also obtained from Cell Signaling Technologies.

### Cell Culture

HCT116 WT and HT-29 (ATCC) as well as HCT116 p53^−/−^ cells were cultured in Dulbecco's modified Eagle medium (DMEM) (Gibco, Germany) supplemented with 10% (v/v) FCS (Gibco) and 1% (v/v) penicillin/streptomycin (Gibco) and incubated at 37°C under 5% CO2 in a humidified atmosphere. The HCT116 p53^−/−^ cell line used in this study was originally introduced by Bunz et al. (20), was a kind gift from Dr. Thomas G. Hofmann (DKFZ, Heidelberg, Germany). Cells were passaged upon reaching 75–80% confluence. After seeding at the desired density, cells were maintained overnight in the incubator before starting the experiments. All the treatments were performed in standard medium containing 10% FCS and 1% antibiotics.

### Cell Viability Assay

Cells were seeded in 96-well plates at a density of 10,000 cells/well and treated with the increasing concentrations of either MC3 (0.25–20 μM) or auranofin (1–20 μM) on the following day. At different time points (24, 48, and 72 h) the medium was replaced with DMEM (1% FCS), containing 0.5 mg/mL MTT. Cells were incubated for 1–2 h and intracellular formazan pellets were solubilized using DMSO (Sigma-Aldrich). Absorbance was subsequently measured at 595 nm using the Tecan Ultra plate reader (Tecan, Germany).

### FACS Analysis of Apoptotic Cell Death

200,000 cells/well were seeded in a 12-well plate and placed overnight in an incubator. Then, the compound was applied to the cells at the indicated concentrations. Cells were harvested 24 h later, and 250,000 cells from each well were collected, washed once with the annexin V binding buffer (Bioscience, Germany), re-suspended in 50 μL binding buffer containing 5 μL FITC-conjugated annexin V (AV, Bioscience) and 5 μL propidium iodide (PI, Bioscience). Samples were then incubated in the dark for 10–15 min at room temperature. Cell suspensions were diluted with 450 μL binding buffer and were analyzed immediately using Guava easyCyte HT sampling flow cytometer (Guava Technologies, Hayward, CA). FITC annexin V and PI were detected at excitation/emission wavelengths of 488/518 and 488/617 nm, respectively. Data were analyzed using the software GuavaSoft 3.1.1 (Guava Technologies).

### Intracellular ROS Formation Assay

HCT116 clones and HT-29 cells, seeded at a density of 200,000 cells/well, were treated with increasing concentrations of MC3 (0.25–5 μM) for either 3 or 24 h. At the end of treatment period, 250,000 cells/well were harvested and re-suspended in phenol red-free DMEM containing 30 μM dihydroeithidium (DHE, Biomol GmbH). After 15 min of incubation in the dark, cellular pellets were re-suspended in 500 μL of DMEM without phenol red. FACS analysis was performed immediately using Guava easyCyte HT sampling flow cytometer at 488 nm excitation and 564–606 nm emission wavelengths. Data were analyzed using the software GuavaSoft 3.1.1.

### Cell Cycle Analysis

500,000 cells/well were seeded in 6-well plates. After incubation overnight, cells were treated with the compound at concentrations of 1 and 5 μM. 24 h later, 10^6^ cells were collected, washed once with PBS, fixed using ice-cold 70% ethanol in a drop-wise manner, and placed overnight at −20°C. Samples were then incubated with RNAase (50 μg/mL) at 37°C for 30 min followed by incubation with PI (100 μg/mL) for 30 min in the dark. Samples were then analyzed by FACSCalibur (Becton Dickinson).

### Mitochondrial Membrane Potential (MMP) Assessment

Flow cytometry analysis of JC1 staining was performed as previously described (21). Briefly, the three CRC cells were incubated with 2 μM of JC1 for 15 min, at the end of the respective treatments. FACS analysis was immediately performed by Guava easyCyte HT sampling flow cytometer. Additionally, MMP was determined using the BIOREVO fluorescence microscope (BZ9000, KEYENCE).

### Mitochondrial Purification for Western Blot Analysis

Mitochondria were isolated from CRC cells by pestle homogenization and differential centrifugation. At the end of treatments, cells were collected and washed three times with NKM buffer (containing 1 mM Tris-HCl, 0.13 M NaCl, 5 mM KCl, and 7.5 mM MgCl_2_). Thereafter, cellular pellets were resuspended in homogenization buffer (10 mM Tris-HCl, 10 mM Kcl, 0.15 mM MgCl_2_ supplemented with 1 mM PMSF and 1 mM DTT) and were incubated for 15 min on ice, then homogenized using a pestle attached to a drill at medium speed for 10–30 passes. Homogenized cells were mixed with a 2 mM solution of sucrose and centrifuged at low speed in order to pellet nuclei, unbroken cells, and large debris. Mitochondria were finally purified by centrifugation at high speed and were kept in mitochondrial suspension buffer (10 mM Tris-HCl, 0.25 mM sucrose, 0.15 mM MgCl_2_ supplemented with 1 mM PMSF and 1 mM DTT). Protein concentration was determined using Bradford reagent (Sigma-Aldrich).

### Western Blot Analysis of Protein Expression

As previously described (22), cells were lysed using a urea-based lysis buffer (6 M urea, 1 mM EDTA, 5 mM NaF, 0.5% Triton X-100) supplemented with multiple protease and phosphatase inhibitors (10 μg/mL pepstatin, 0.1 mM PMSF, 10 μg/mL aprotinin, 1 mM sodium orthovanadate, and 2.5 mM sodium pyrophosphate). Protein concentration was determined using Bradford reagent. After denaturation at 95°C for 5 min, 50 μg of the total protein per sample was resolved by SDS-PAGE and then transferred onto a PVDF membrane (GE healthcare, Germany), and blocked with 5% (w/v) milk in TBS/Tween for 2 h at room temperature. Membranes were subsequently incubated overnight with the desired primary antibodies at 4°C. After washing with TBS/Tween, membranes were further incubated for 1 h at room temperature with the corresponding anti- mouse or -rabbit IgG HRP-conjugated secondary antibodies dissolved in 5% milk in TBS/Tween. Target proteins were finally visualized using the HRP substrate, Western Lightning Plus-ECl (Perkin Elmer), and were imaged by the Fujifilm LAS-3000 imaging system.

### Immunofluorescence Microscopy

The human CRC cell lines were seeded on a Ø-12mm cover slip coated with Geltrex (Life Technologies, Germany) and placed overnight in an incubator, and then treated with MC3 or its vehicle (0.1% (v/v) DMF). At the end of treatments, cells were fixed using 4% PFA for 10–15 min at room temperature, and then washed at least two times with PBS and incubated for 30 min with the blocking buffer (5% goat serum, 1% BSA, and 0.3% Triton X-100 in PBS). Cells were subsequently incubated with the antibody solutions prepared in the blocking buffer and were incubated overnight at 4°C with gentle agitation. The antibody solutions were then replaced with the matching goat anti- rabbit (Alexa Flor 488) or -mouse (Alexa Flor 594, Dianova, Germany) secondary antibodies together with Hoechst 33342 (1 μg/mL, Sigma-Aldrich) in order to indicate nuclei. After 1 h of incubation, cells were washed sufficiently with PBS and were imaged using the BIOREVO fluorescence microscope (BZ9000, KEYENCE).

### RNA Isolation, Reverse Transcription and Quantitative Real-Time PCR

After the aforementioned treatments with MC3 (1 and 5 μM) or the vehicle, total RNA was extracted from the cells, using the QIAzol lysis reagent (Qiagen, Germany). The concentration and purity of the isolated RNA were determined by the NanoDrop 2000 spectrophotometer (Thermo Scientific, Germany). cDNA was synthesized by reverse transcription of 500 ng of RNA using the ProtoScript® II first strand cDNA synthesis kit (NEB). The mRNA expression of the desired genes was analyzed by real-time thermal cycler q-Tower (Analytik Jena, Germany), using the reaction mix LightCycler® 480 SYBR Green I Master (Roche, Germany) and the following primer sequences (Eurofins, Germany): *CDKN1A* (5s: GACACCACTGGAGGGTGACT; 3as: CAGGTCCACATGGTCTTCCT), *TP53* (5s: CCTCACCATCATCACACTGGAAG; 3as: CCTTTCTTGCGGAGATTCTCTTCC), *TP73* (5s: CATGGAGACGAGGACACGTA; 3as: GTGACTCGGCCTCTGTAGGA), *Bax* (5s: GGGGACGAACTGGACAGTAA; 3as: CAGTTGAAGTTGCCGTCAGA), *Noxa* (5s: CTGGACAAAAGCGTGGTCTC; 3as: GCGAGCTGAACACGAACAGT), *Puma* (5s: GACGACCTCAACGCACAGTA; 3as: CACCTAATTGGGCTCCATCT), *Actin* (5s: CTGACTACCTCATGAAGATCCTC; 3as: CATTGCCAATGGTGATGACCTG).

### Transient Transfection Studies

HCT116 p53^−/−^ cells were plated in 96-well plates so they will be at 70–90% confluence at the time of transfection. DNA-lipid complexes were prepared in 10 μL/well Opti-MEM reduced serum medium (Gibco), using 0.1 μg/well plasmid DNA and 0.2 μL/well of P3000 and Lipofectamine 3000 reagents (Thermo Fischer, Germany). The mixture was incubated for 15 min at room temperature after which was diluted (1:5) with antibiotic-free DMEM and added to the cells. 24 h later, media was refreshed with the treatments of MC3 (0.2 μM) or its vehicle for 24 h, after which MTT assay was performed. The following constructs carrying either WT p53 or different mutations of *TP53* were used: GFP-p53 (Addgene plasmid # 12091); pcDNA3 p53 S15D (# 69005); pcDNA3 p53 S15A (# 69004); pCMV-Neo-Bam p53 R175H (# 16436), and pCMV-Neo-Bam p53 R273H (# 16439). In all transfections the corresponding empty vectors were used as negative controls and the p53 expression was determined by immunoblotting, except for the GFP-p53 plasmid, where p53 expression was evaluated by the GFP-expressing population of cells using fluorescence microscopy.

In order to knock down the expression of *TP73* and *CDKN1A* (*p21*) genes, transient RNA interference was performed. Briefly, 40 pmol of small interfering (si) RNA against each of the genes and 1.5 μL/well Lipofectamine 3000 were diluted in 100 μL/well of Opti-MEM in a 24-well plate followed by 15 min incubation at room temperature. Cell suspension was then added at a density of 60,000 cells/well. After overnight incubation, cells were treated with fresh media containing MC3 or the vehicle. Anti- p73 and p21 siRNAs were obtained from Thermo Fisher Scientific. Non-targeting siRNA (Thermo Fisher Scientific) was used as the negative control.

### Statistical Analyses

Microsoft Excel and GraphPad Prism were used for statistical analyses. Multiple comparisons were performed using two-way ANOVA followed by a *post-hoc* Tukey test. *p*-values ≤ 0.05, 0.01, 0.001, and 0.0001 determined the statistical significance and are shown in figures as ^*^, ^**^, ^***^, and ^****^, respectively.

## Results

### MC3 Exhibits Cytotoxic Effects Across CRC Cell Lines Harboring Different p53 Profiles

**Figure 1:**
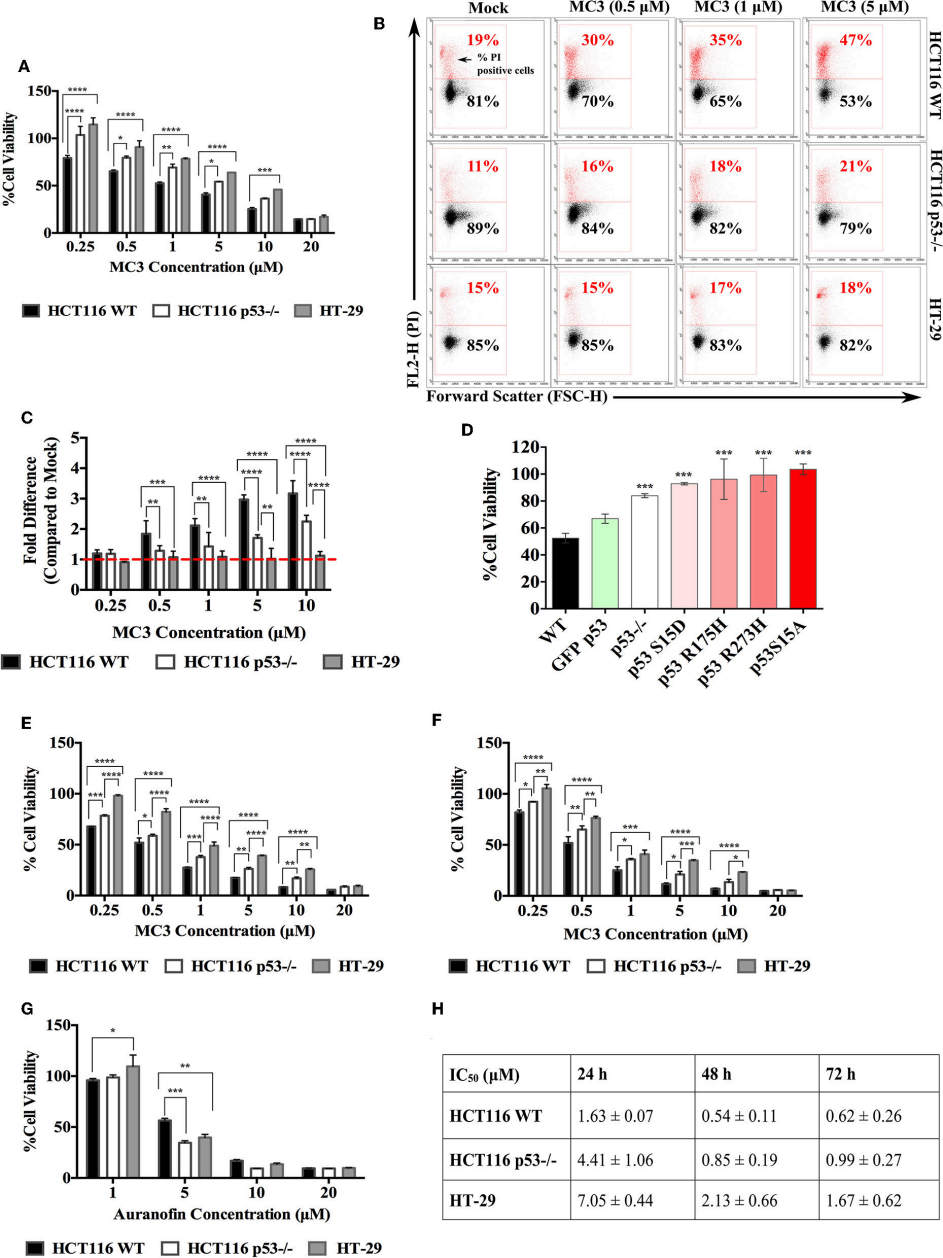
CRC cells harboring WT p53 are more susceptible to the anti-proliferative effects of MC3. **(A)** CRC cell lines with different p53 statuses were treated with increasing concentrations of MC3. After 24 h, MTT viability assay was performed. A p53-dependent response to MC3 was observed, with HT-29 being the most resistant cell line to the drug's cytotoxic effect in most of the tested concentrations. Cell viability was determined as the number of treated over control (0.1% DMF) cells. **(B)** Representative density plots from FACS analysis show a higher percentage of PI positive cells in HCT116 WT upon 24 h of MC3 treatment in comparison to that of HCT116 p53^−/−^ and HT-29 at most tested conditions. 0.1% DMF was used as mock. **(C)** The fold change of PI positive cells (normalized to mock) significantly decreases in cells lacking WT p53 after 24 h of MC3 treatment. **(D)** HCT116 p53^−/−^ cells were transfected with over-expression plasmids carrying different p53 mutations in a number of hotspots as indicated, or GFP-tagged WT p53. 24 h later, cells were treated with 0.2 μM of MC3, and MTT assay was employed after 24 h of treatment. Cell viability was determined as the number of treated over control (0.1% DMF) cells. Asterisks show significance in survival percentages of HCT116 p53^−/−^ and transfected cells vs. HCT116 WT, calculated by two-tailed student's *t*-test. **(E,F)** HCT116 WT and its p53^−/−^ clone as well as HT-29 were treated with increasing concentrations of MC3 for 48 h **(E)** and 72 h **(F)**, after which cell viability was determined using MTT assay. 0.1% DMF was used as mock. **(G)** The anti-proliferative effect of auranofin occurs independent of p53 status in CRC cell lines. Cellular survival of HCT116 WT and p53-deficient as well as HT-29 cells treated with increasing concentrations of auranofin for 24 h, determined by MTT assay. Cell viability was measured as the number of treated over control (0.1% DMSO) cells. The cytotoxic efficacy of auranofn is not significantly higher in the presence of WT p53 at most of the tested concentrations. **(H)** IC_50_ values of MC3 in the three CRC cell lines at the time points indicated. Multiple comparisons were employed using two-way ANOVA test and a *post-hoc* Tukey test. The results in **(A,C–H)** are mean ± SD from at least three independent experiments, one is shown in **(B)**. *, **, ****, and **** represent *p*-values ≤ 0.05, 0.01, 0.001, and 0.0001, respectively.

In our previous report (18) MC3 showed strong anti-proliferative and pro-apoptotic effects in pancreatic cancer cells namely Bxpc3 (mutant p53; Y220C), Miapaca2 (R248W), the gemcitabine-resistant ASPC1 (R273H) as well as Panc1 (R273H). For the initial experiment, we sought to confirm these effects in the CRC cell lines HCT116 WT, HCT116 p53^−/−^, and HT-29. The anti-proliferative effects of MC3 were determined by MTT assay and PI staining. After 24 h of treatment, we found significantly lower cell viabilities in HCT116 WT compared with those of the other two cell lines at most tested concentrations (Figure 1A). Additionally, treatment led to increased population of PI positive cells in HCT116 WT and p53^−/−^, however with a significantly higher efficacy in the presence of WT p53. In the case of HT-29, the number of dead cells was non-significantly altered (Figures 1B,C).

Most of the *TP53* mutations cluster within the central DNA binding domain of the protein and a number of hotspots have been identified in this region (8). Different mutations give rise to mutant p53 proteins, that show different degrees of functionality and biological activities (8). To further evaluate the p53-dependent response of CRC cells to MC3, we broadened our investigation to encompass more p53 variations. To this end, different constructs with DNA-contact mutations (R273H, S15A, and S15D), the structural mutant R175H as well as WT p53 were transiently over-expressed in HCT116 p53^−/−^ and cell viability was determined after 24 h of MC3 treatment. Transient over-expression of different mutant p53-harboring constructs attenuated the cytotoxicity of the molecule (Figure 1D).

A time dependent MTT assay was also performed to assess the long-term effects of MC3 on the three CRC cell lines. As expected, the cytotoxic effect of MC3 was enhanced over time, with HT-29 remaining the most resistant cell line (Figures 1E,F,H) and Supplementary Figure 1). As a reference, the cytotoxicity of auranofin was tested in HCT116 and HT-29 cells. In contrast to MC3, the toxicity of auranofin occurred irrespective of p53 status (Figure 1G). This finding is supported by previous reports, demonstrating a p53-independent mode of action for auranofin (16, 17).

### Intracellular ROS Production Is Elevated in Response to MC3

**Figure 2:**
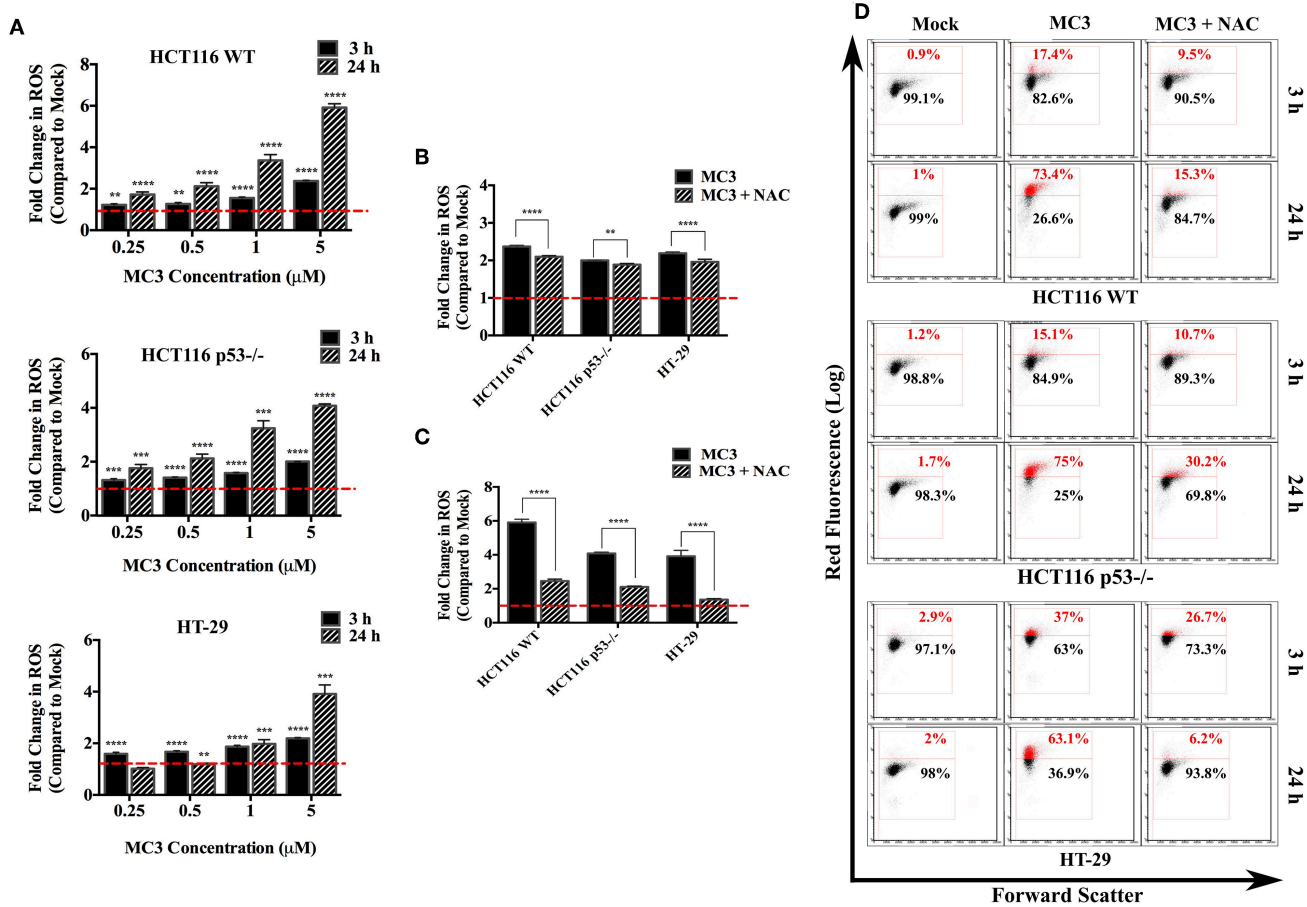
Intracellular ROS levels are induced by MC3 in CRC cells. **(A)** CRC cell lines were treated with MC3 in a concentration-dependent manner as indicated, for either 3 or 24 h, thereafter ROS generation was analyzed by FACS using the dye, DHE. Data were normalized to the respective mock (0.1% DMF) treatment. Statistical comparison was made between each concentration and mock using two-tailed student's *t*-test. **(B,C)** The ROS scavenger, NAC significantly attenuates the induction in intracellular ROS levels with MC3 treatment (5 μM) in all three cell lines after 3 h **(B)** and 24 h **(C)** time points. Comparison of the fold difference in ROS production between NAC-treated and -untreated cells was performed using two-tailed student's *t*-test. **(D)** Representative density plots of one out of four biological replicates shown in **(A–C)**. Error bars ± SD; *n* = 4. **, ***, and **** denote *p*-values ≤ 0.01, 0.001, and 0.0001, respectively.

TrxR inhibition by gold complexes, such as MC3 decreases the levels of reduced Trx, which has a central role in eliminating intracellular ROS (23). On the other hand, p53 has been shown to influence the intracellular ROS balance, with target genes, such as *PIG-1-13, Bax*, and *Puma* stimulating ROS production, whereas others (e.g., TIGAR) contributing to cellular anti-oxidant defense (19, 21). We therefore questioned if p53 status impacts the ROS inducing ability of MC3. While a significant increase in intracellular ROS levels was detected in the three CRC cell lines as well as p53 R273H over-expressing HCT116 upon MC3 treatment (Figure 2A and Supplementary Figure 2A), HCT116 WT cells were found to exhibit higher ROS induction compared to the other two cell lines after long-term exposure to the highest concentration of MC3 (5 μM) (Figures 2A–C). Additionally, we pre-incubated the three cell lines with NAC (10 mM) 1 h prior to the addition of MC3 to assess the role of anti-oxidant treatment in neutralizing intracellular ROS accumulation caused by the molecule. As shown in Figures 2B–D and Supplementary Figure 2A, NAC led to a significant reduction in intracellular ROS in all cell lines.

### MC3 Induces p21 Expression and Inhibits Cell Cycle Progression in CRC Cells

**Figure 3:**
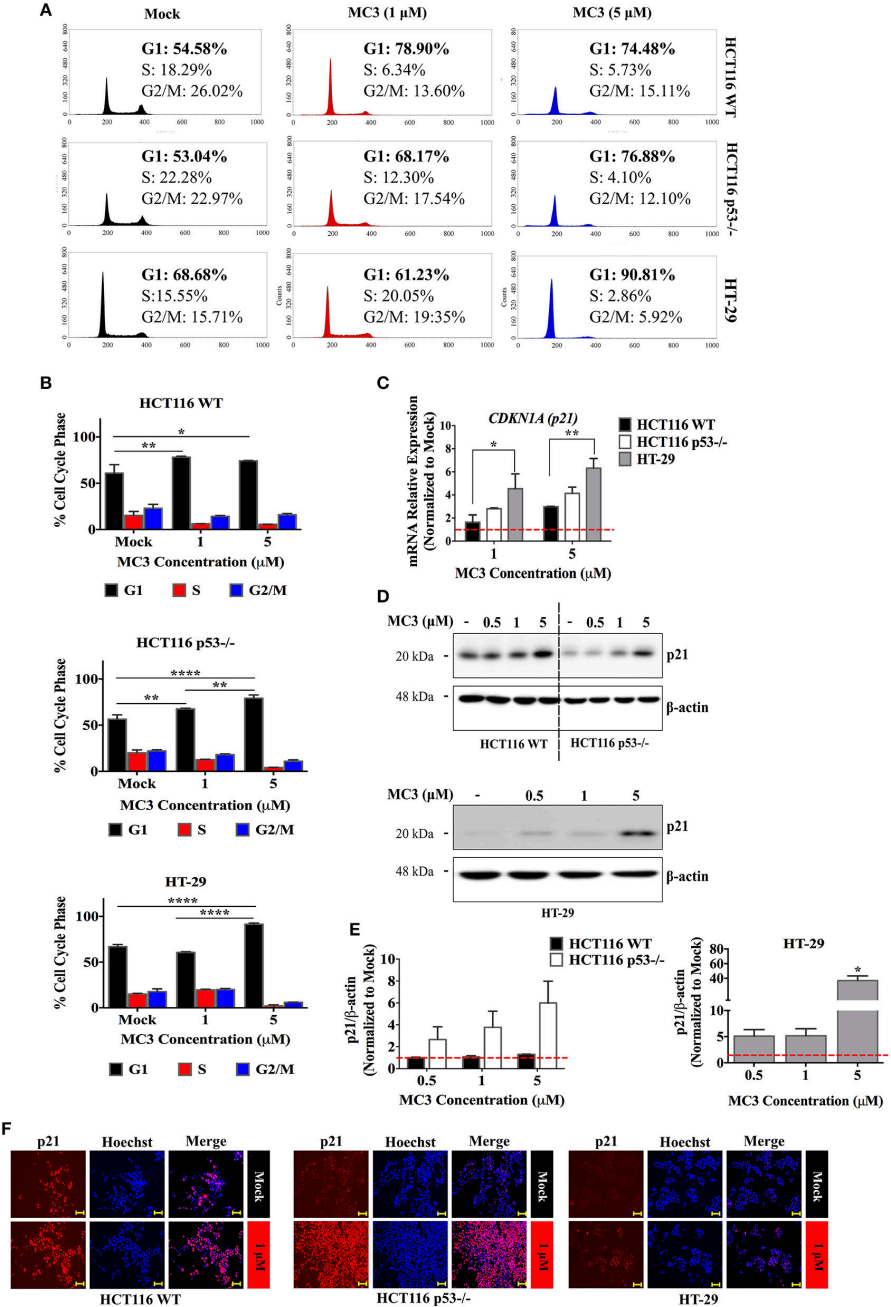
MC3 treatment strongly activates the CDK inhibitor, p21 and prolongs the G1/S cell cycle transition in CRC cell lines. **(A)** Representative histogram plots illustrate changes in the cell cycle phase distribution upon 24 h of MC3 treatment (1 and 5 μM) across all three cell lines. **(B)** A G1 phase arrest was observed in both clones of HCT116 after 24 h of MC3 treatment at both tested concentrations (1 and 5 μM) but was seen only with the higher concentration (5 μM) in HT-29 cells. The results are obtained from two independent experiments, one of which is depicted in **(A)**. Error bars ± SD. **(C)** The regulation of *p21* (*CDKN1A*) mRNA expression after 24 h treatment with MC3 (1 and 5 μM) was found to be p53 dependent, with HT-29, having the highest induction compared to mock. Data present mean ± SD from at least three biological replicates. β-actin served as a housekeeping gene. **(D)** MC3 induces the expression of p21, in the three CRC cell lines upon 24 h treatment in a concentration-dependent manner as indicated, determined by immunoblotting. β-actin was used as the loading control. **(E)** Densitometric analysis of p21 bands normalized to those of β-actin, normalized to the respective mock treatment. Error bars represent the SEM of two biological replicates, one of which is shown in **(D)**. **(F)** Immunocytochemistry investigations demonstrating p21 nuclear localization in the three cell lines in response to MC3 (1 μM, 24h), with the induction–compared to the basal levels–being more pronounced in cells lacking WT p53. Hoechst dye was used to indicate nuclei. Scale bar: 100 μm. 0.1% DMF was used as mock treatment. Multiple comparisons were employed using two-way ANOVA test and a *post-hoc* Tukey test. *, **, and **** denote *p*-values ≤ 0.05, 0.01, and 0.0001, respectively.

Excessive ROS is known to trigger replication stress and subsequently the DNA damage response (DDR) (24). p53 is a well-known component of DDR, which induces cell cycle arrest, apoptosis or senescence by transcriptional regulation of several genes, among others, *CDKN1A* (*p21*) and the Bcl-2 family members, *Bax* and *Puma* (25). Analysis of DNA content revealed a G1/S arrest with 24 h of MC3 treatment (1 μM and 5 μM) in HCT116 WT and p53^−/−^ as well as in HT-29 cells treated with 5 μM of MC3 (Figures 3A,B).

The CDK inhibitor, p21 is a direct transcriptional target of p53, although it can be regulated by several p53-independent pathways (26). Given the clear cell cycle inhibition in CRC cells by MC3, we investigated the influence of treatment on the mRNA and protein expression of p21. 24 h of treatment with MC3 (5 μM) led to a clear induction in p21 mRNA and protein levels in all three cell lines as well as HCT116 p53^−/−^ cells over-expressing R273H mutation (Figures 3C–E and Supplementary Figure 2B). Noteworthy is that basal p21 levels appeared to correlate with p53 status, where WT cells exhibited higher p21 protein levels compared to p53-negative and mutant p53-harboring cells (Figure 3D and Supplementary Figure 2B).

Several lines of evidence have suggested that the primary role of p21 in cellular growth and survival depends on its subcellular compartmentalization. While nuclear p21 favors its tumor suppressive functions, cytoplasmic p21 has been associated with oncogenic properties, such as inhibition of apoptosis as well as enhanced cellular proliferation and migration (27). In this regard, we sought to determine the cellular localization of p21 by immunocytochemistry. p21 was found to localize in the nucleus in all the CRC cell lines treated with MC3 (Figure 3F). Altogether, these findings demonstrate that MC3 activates cell cycle checkpoints, such as p21, subsequently leading to proliferation arrest in CRC cells possessing distinct p53 variations.

### MC3 Triggers Disparate Apoptotic Markers in WT and p53-Null HCT116 Cells

**Figure 4:**
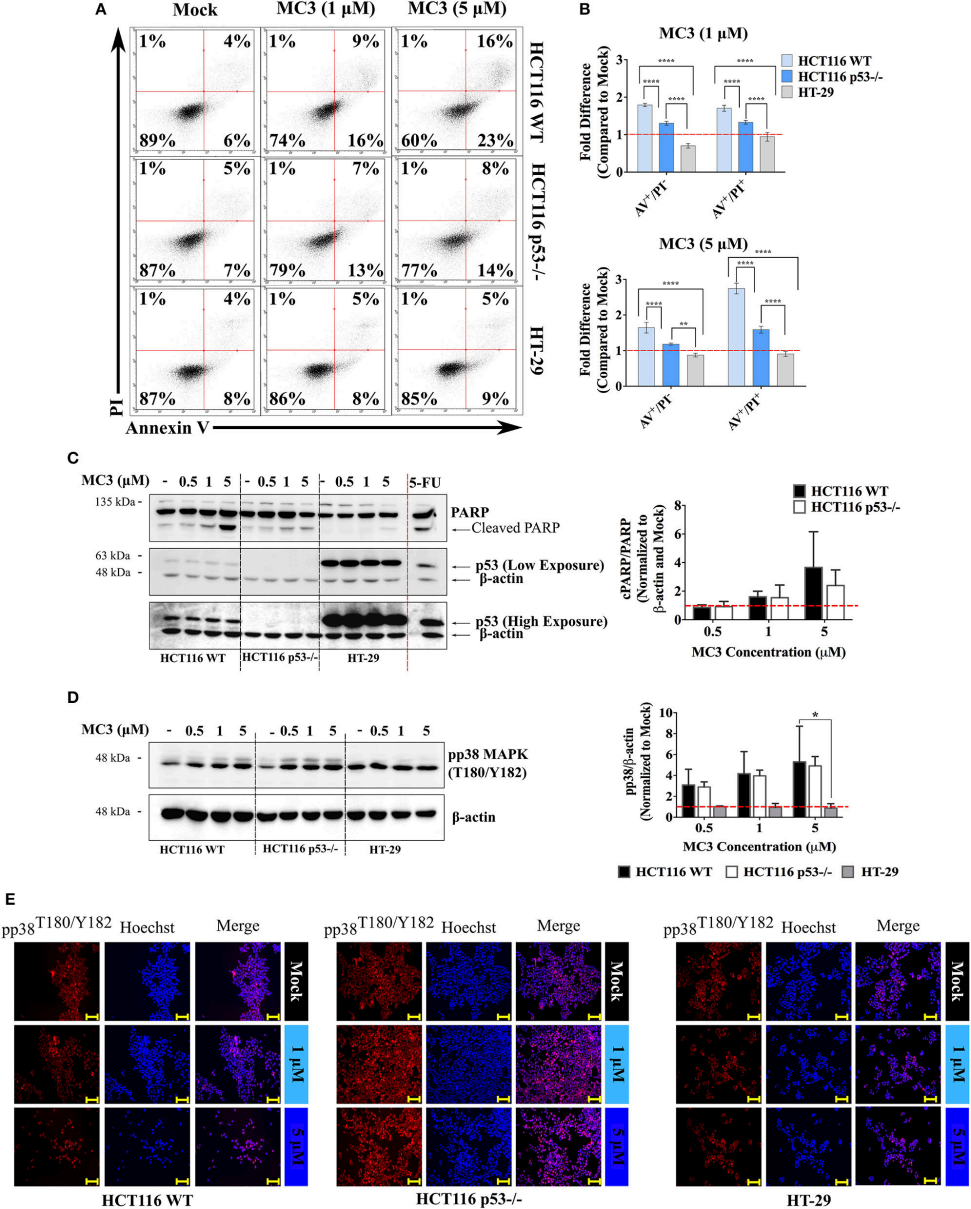
MC3 differentially activates apoptotic markers across CRC cell lines. **(A)** Representative AV/PI graphs of CRC cells treated with 1 and 5 μM of MC3 for 24 h. Data show transition of HCT116 clones through the AV^+^/PI^−^ and AV^+^/PI^+^ quadrants, an effect which is not detected in mutant p53-harboring HT-29 cells. **(B)** MC3 induces apoptotic/necrotic cell death in the isogenic HCT116 cell lines after 24 h at both tested concentrations (1 and 5 μM) as determined by annexin V/PI staining, however with a significantly higher efficacy in the presence of WT p53. Data represent mean ± SD from at least four biological replicates, one of which is depicted in **(A)**. **(C)** and **(D)** Protein levels of cleaved PARP, a hall mark of apoptosis, as well as the pro-apoptotic signaling molecule, pp38 MAPK (T180/Y182) are induced by MC3 (24 h) in a concentration-dependent manner in HCT116 WT and p53^−/−^, but not in HT-29 cells. β-actin served as the loading control. HCT116 WT cells were treated with a concentration of 100 μM of 5-FU for 24 h as a positive control for PARP cleavage. Bar graphs show densitometric analyses of cPARP/PARP and pp38 MAPK (T180/Y182) bands normalized to their respective loading controls and mock treatments. Error bars are the SEM of two independent experiments, one immunoblot of which is presented. Multiple comparisons were performed using two-way ANOVA test and a *post-hoc* Tukey test. *, **, and **** denote *p*-values ≤ 0.05, 0.01, and 0.0001, respectively. **(E)** Consistent with immunoblotting, MC3 induces pp38 MAPK (T180/Y182) nuclear accumulation in WT and p53-deficient HCT116 cells, but not in HT-29. Cells were treated with various concentrations of MC3 as indicated for 24 h, after which immunocytochemistry was employed using specific antibodies. Hoechst dye was used to visualize nuclei. Scale bar: 200 μm. Vehicle (0.1% DMF)-treated cells served as mock.

p53 induces ROS-associated apoptosis via both intrinsic and extrinsic pathways (28). MC3-mediated apoptosis in pancreatic cancer cells has been previously linked to its elevation of ROS levels, leading to oxidative stress-related cell death (18). We therefore investigated the apoptotic response to MC3 and its relevance to p53 status in the three CRC cell lines. One of the hallmarks of early apoptosis is externalization of phosphatidylserine on the cell membrane that precedes loss of membrane integrity and can be monitored by AV/PI staining (18). As shown in Figure 4A, a 24 h treatment with MC3 (5 μM) resulted in ~40% and ~23% loss of cell viability in HCT116 WT and p53^−/−^, respectively, with cells progressing to the AV^+^/PI^−^ and AV^+^/PI^+^ quadrants. This is consistent with our initial experiment that showed a p53-dependent cytotoxic response to MC3 (Figures 1A–C). In contrast to this, MC3 was unable to affect cell viability in mutant p53-harboring HT-29 cells (Figures 4A,B). Using immunoblotting, we further analyzed the occurrence of apoptotic cell death by measuring the activation of p38 MAPK as well as poly ADP-ribose polymerase (PARP) cleavage. After 24 h of treatment, a concentration dependent accumulation of phospho-p38 MAPK (pp38 MAPK; T180/Y182) and cleavage of PARP were observed in the two HCT116 clones, however were hardly detectable in HT-29 and HCT116 p53 R273H cells (Figures 4C,D and Supplementary Figure 2B). The above findings are further supported by the immunofluorescent-based detection of nuclear pp38 MAPK (T180/Y182) accumulation in both HCT116 clones but not in HT-29 (Figure 4E).

**Figure 5:**
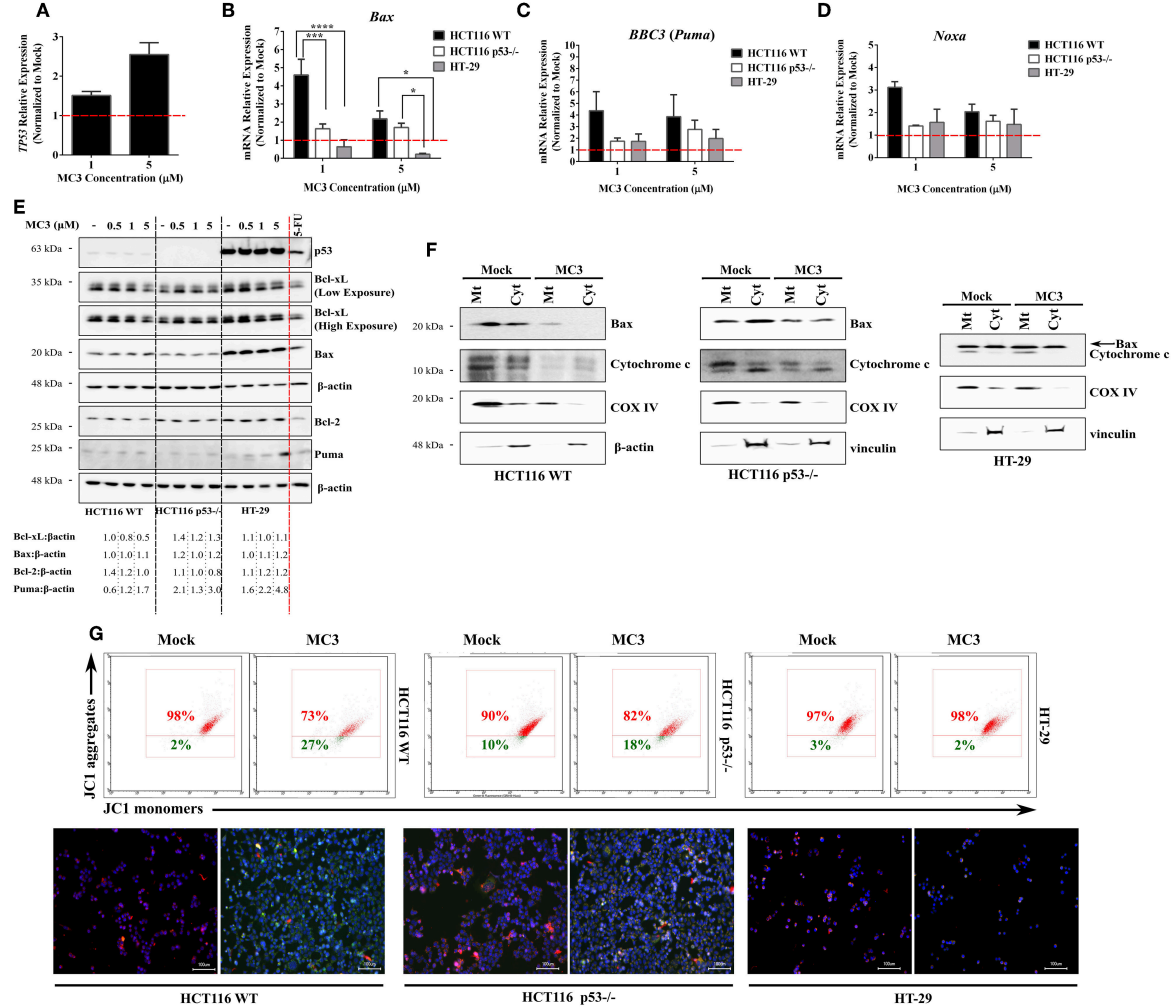
MC3 differentially regulates the expression of pro-apoptotic Bcl-2 family members in the different CRC cells. **(A–D)** qRT-PCR analysis of the mRNA expression of *TP53* and its pro-apoptotic targets of the Bcl-2 family in CRC cells treated for 24 h with 1 and 5 μM of MC3. Induction in the transcript levels of *Bax, BBC3* (*Puma*) and *Noxa* by MC3 appeared to be more potent in HCT116 WT cells. mRNA relative expression was calculated by the ΔΔ Ct method where the Ct values of the desired genes were normalized to those of the housekeeping gene (β-actin). Data represent mean ± SEM of at least three biological replicates. Multiple comparisons were employed using two-way ANOVA test and a *post-hoc* Tukey test. *, ***, and **** are representative of *p*-values ≤ 0.05, 0.001, and 0.0001, respectively. **(E)** 24 h of treatment with various concentrations of MC3 as indicated decreases the Bcl-xL protein expression in WT p53-expressing HCT116 cells, but not in cells with p53 mutation or deficiency. The multi-domain member, Bax protein expression is only mildly altered by MC3 in the three cell lines. On the other hand, the BH3-only protein, Puma was found to be markedly activated in HT-29 cells in response to MC3. HCT116 WT cells were treated with 5-FU (100 μM) for 24 h as a positive control. Numbers below show values obtained from densitometric analyses of the presented blots normalized to the respective loading controls (β-actin) and the corresponding mock (0.1% DMF) treatments. **(F)** Mitochondrial (Mt) and cytosolic (Cyt) fractions of the three CRC cells were isolated in order to assess the release of cytochrome *c* as well as subcellular localization of Bax. Cells were either treated with 0.1% DMF (as mock) or 5 μM of MC3 for 24 h, after which subcellular fractionation was performed. Cytochrome *c* oxidase subunit IV (COX IV) was used as mitochondrial loading control, whereas either β-actin or vinculin served as cytosolic controls. **(G)** MC3 (5 μM, 24 h) induces mitochondrial membrane depolarization in the HCT116 clones, but not in HT-29, as evaluated by flow cytometry analysis- and immunofluorescence of JC1 staining. Hoechst dye was used to indicate nuclei. Scale bar: 100 μm.

The intrinsic apoptotic pathway is triggered upon an imbalance of pro- and anti- apoptotic Bcl-2 family members, which results in permeabilization and activation of caspases (28). We therefore investigated the effects of MC3 on the transcript levels of selected members of this family and observed a p53-dependent regulation of their expression. Consistent with the induction of *TP53* mRNA levels in HCT116 WT cells with MC3 treatment (Figure 5A), pro-apoptotic Bcl-2 family members/p53 target genes–*Bax, Noxa* and *BBC3* (*Puma*)–were also found to be up-regulated. This effect was attenuated in p53-null and mutant p53-harboring CRC cells (Figures 5B–D). Interestingly, MC3's induction of Puma's protein expression was highest in HT-29 cells, followed by HCT116 p53^−/−^ cells, and then HCT116 WT (Figure 5E). With regards to the pro-survival Bcl-2 family member Bcl-xL, MC3 was found to reduce its protein levels in HCT116 WT, whereas its expression in p53^−/−^ and HT-29 cells either remained stable or mildly increased (Figure 5E). Moreover, in contrast to its mRNA expression, no clear differences were observed in Bax protein levels with MC3 treatment (Figures 5B,E). Additionally, we did not observe a significant effect of treatment on p53 protein levels or nuclear accumulation in HCT116 WT cells, despite the induction in its transcriptional activity (Supplementary Figure 3). Similarly, p53 protein expression and nuclear localization in HT-29 cells appeared to be unaffected by MC3 (Supplementary Figure 3).

To further assess the involvement of mitochondrial apoptotic pathway in MC3's mediated cell death, we performed subcellular fractionation and looked at the release of cytochrome *c* as well as the cellular localization of Bax (Figure 5F). With regards to the former, HCT116 WT demonstrated a clear accumulation of cytochrome *c* in the cytosolic fraction in MC3-treated cells. This effect was found to be attenuated in p53^−/−^ cells, albeit a clear decrease in the mitochondrial/cytoplasmic ratio of cytochrome c in treated vs. non-treated cells. In case of the latter, Bax protein levels were found to be mostly confined to the mitochondrial fraction in MC3-treated cells as compared to that of mock treatment in HCT116 WT, and to a lesser extent in p53-null HCT116 cells. HT-29, on the other hand, did not show a significant change neither in Bax localization nor with regards to cytochrome *c* release. As aforementioned, intrinsic apoptotic pathway is normally associated with a decrease in MMP, leading to permeabilization. In view of this, MC3 induced mitochondrial membrane depolarization in HCT116 clones, with WT cells showing higher sensitivity. Consistent with the lack of apoptosis, HT-29 cells' MMP was found to be mostly unchanged in response to MC3 (Figure 5G).

### The Anti-Tumor Effect of MC3 in CRC Cell Lines Is Independent of the p53 Homolog, p73

**Figure 6:**
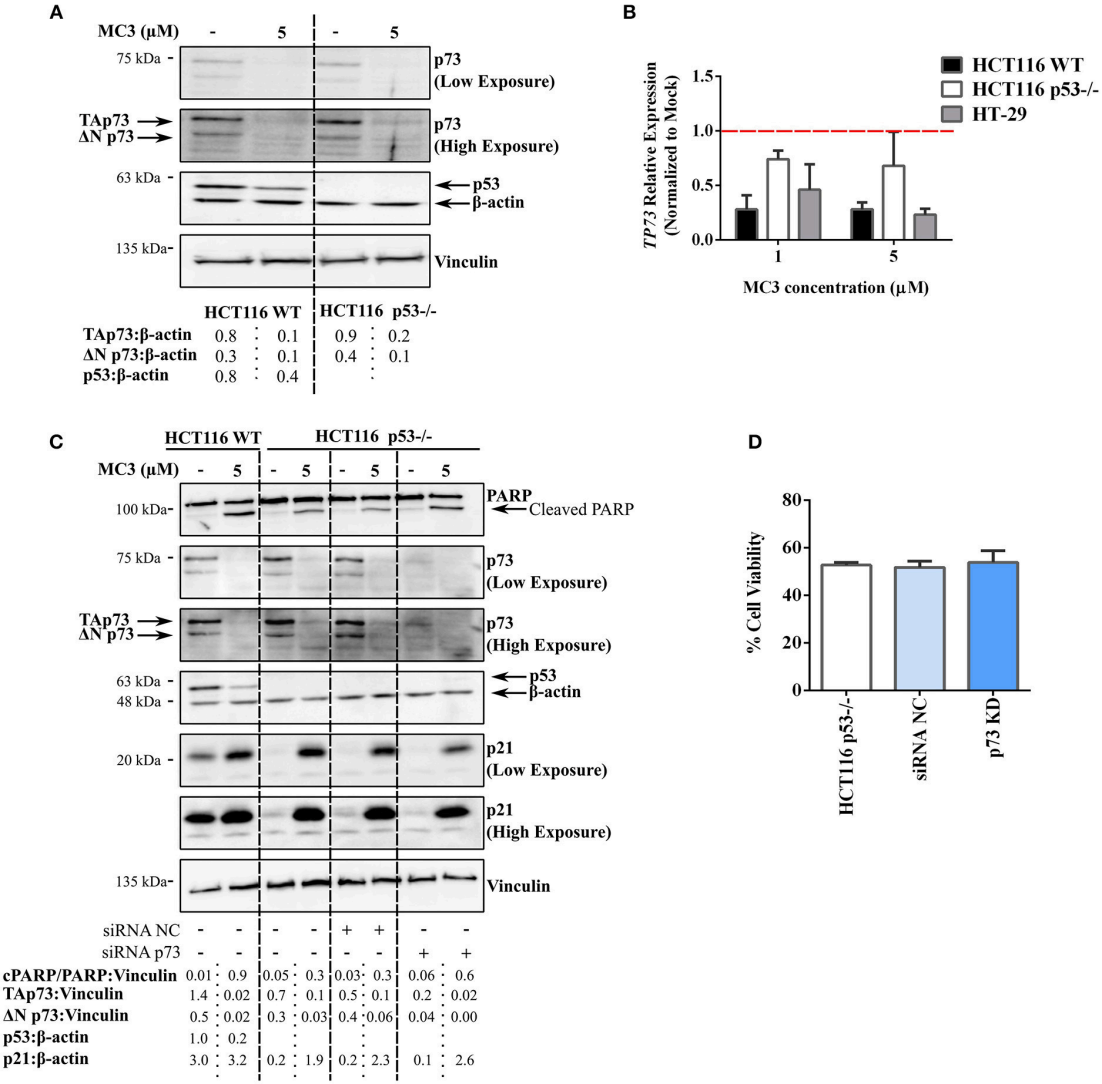
MC3-indued cytotoxicity appears to be independent of the tumor suppressor, TAp73. **(A)** Western blot analysis reveals a strong reduction in the two major isoforms of p73, TAp73 and ΔNp73, in WT and p53^−/−^ HCT116 cells treated with either 5 μM of MC3 or the vehicle (0.1% DMF) for 24 h. β-actin and vinculin were used as loading controls. Numbers show values obtained from densitometric analyses of the presented blots normalized to the respective loading controls (β-actin). **(B)** 24 h of treatment with MC3 at the indicated concentrations down-regulates the *TP73* gene in the three CRC cell lines. mRNA levels were analyzed by qRT-PCR and the relative gene expression was determined using the ΔΔ Ct method where the Ct values of *TP73* were normalized to those of β-actin. 0.1% DMF was used as mock. Error bars indicate the SEM of three biological replicates. **(C)** Knock-down of p73 in HCT116 p53^−/−^ does not hamper MC3-induced PARP degradation as well as p21 induction. p53-deficient HCT116 cells were transfected with anti-p73 siRNA (HCT116 p53^−/−^p73KD) or a negative control (HCT116 p53^−/−^ siRNA NC), thereafter were treated with 0.1% DMF or MC3 (5 μM) for 24 h. Transfection efficiency was confirmed by a clear reduction in p73 protein expression. β-actin and vinculin served as loading controls. Numbers represent densitometric analyses of the presented blots normalized to the respective loading controls (vinculin). **(D)** Viability of HCT116 p53^−/−^p73KD cells after 24 h treatment with 5 μM of MC3 is reduced in a similar manner to HCT116 p53^−/−^siRNA NC as well as the parental cell line, measured by MTT assay. Percentage cell viability was determined by normalizing the results of MC3-treated cells to those of vehicle (0.1% DMF)-treated ones. Data represent mean ± SD from two independent experiments, each was done in triplicates.

The above-mentioned results clearly implicate the ability of MC3 to up-regulate p53-family target genes (e.g., p21) across the three cell lines regardless of p53 status. Furthermore, pro-apoptotic molecules, such as PARP cleavage, pp38 MAPK (T180/Y182) as well as Bcl-2 family members were induced in both WT and p53-deficient HCT116 cells. We therefore questioned if a p53-related protein, such as p73 or p63, is involved in the anti-tumor responses triggered by MC3. We have shown in a previous study that p73 substitutes p53 in p53-deficient cells, thus influencing the chemo-sensitivity of such cancer cells (29). Since the transactivating (TA) isoform of p63 is rarely expressed in tissues other than germ cells of the ovary and testis (29, 30), we focused in the present report on the potential role of p73. Surprisingly, a 24 h treatment with 5 μM of MC3 led to a significant depletion in p73 protein and mRNA levels in both HCT116 cell lines (Figures 6A,B). p73 is expressed as multiple isoforms, namely the TA (TAp73) and ΔNp73 isoforms, with opposing effects on apoptosis and the cell cycle (31). The latter not only lacks the transcriptional activity, but may also act as an inhibitor of the tumor suppressors, TAp73 and p53 (31). In this regard, MC3 was found to reduce the expression of both isoforms (Figures 6A,C). To confirm the lack of involvement of p73 in MC3's responses, p73 levels were transiently knocked down (KD) in HCT116 p53^−/−^ cells using siRNA. HCT116 p53^−/−^p73KD cells were subsequently treated with 5 μM of MC3 for 24 h, after which PARP cleavage and p21 levels were analyzed by immunoblotting and cell viability by MTT assay. As depicted in Figure 6C, MC3-induced apoptosis was not attenuated in HCT116 p53^−/−^p73KD cells since the cleavage of PARP was clearly detectable. Furthermore, knocking down p73 did not dampen MC3's induction of p21 levels (Figure 6C) and did not significantly influence the molecule's effect on cell viability (Figure 6D). These data suggest a TAp73-independent mode of action of MC3 in p53-deficient cells, in which apoptosis and growth-inhibitory effects are induced by alternative pathways.

### Modulation of the Cell Cycle Regulator, p21 Influences the Cellular Survival of p53-Deficient HCT116 Cells in Response to MC3

**Figure 7:**
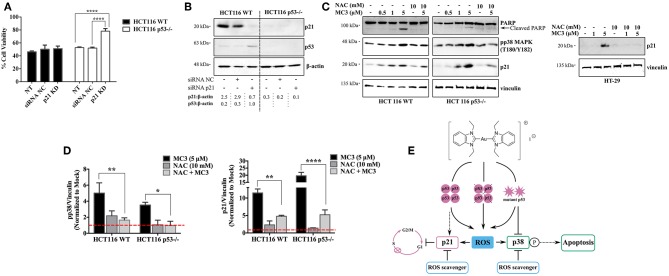
Dual absence of p53 and p21 causes resistance to the MC3-mediated cytotoxicity. **(A)** HCT116 WT and p53^−/−^ cells were transfected with either NC- or anti-p21 siRNA and treated with a concentration of 5 μM of MC3 for 24 h, after which cell viability was assessed using MTT assay. Error bars represent the SD from two independent experiments, each was done in triplicates. **(B)** Confirmation of p21 knock-down determined by immunoblotting. Numbers denote values obtained from densitometric analyses of the presented blots normalized to the respective loading controls (β-actin). **(C)** The ROS scavenger, NAC attenuates the MC3-triggered p21 induction in the three CRC cells, as well as pp38 MAPK (T180/Y182) levels and PARP cleavage in the HCT116 clones, detected by immunoblotting. Cells were treated with increasing concentration of MC3 as indicated. NAC (10 mM) was added 1 h prior to the addition of MC3 at the concentrations indicated. **(D)** Densitometric analyses show a significant reduction in MC3-induced pp38 and p21 protein levels in the presence of anti-oxidant treatment. Error bars represent the SEM of four biological replicates, one of which is depicted in **(C)**. Vinculin served as the loading control. 0.1% DMF was used as mock treatment. Multiple comparisons were performed using two-way ANOVA and a *post-hoc* Tukey test. **(E)** Schematic overview of the small molecule's anti-proliferative and pro-apoptotic actions in CRC cells with different p53 backgrounds. *, **, and **** are representative of *p*-values ≤ 0.05, 0.01, and 0.0001, respectively.

Since p21 is a critical mediator for p53-dependent tumor suppressive functions (20, 26, 32), we aimed to verify its role in MC3-mediated cytotoxicity. We therefore knocked down its expression with siRNA in both HCT116 clones (Figure 7B), and then treated cells with MC3 (5 μM) for 24 h, after which MTT assay was performed. As shown in Figure 7A, silencing *p21* did not alter MC3-induced cytotoxicity in the parental cell line, however, it led to enhanced cell viability in the p53-deficient counterpart.

Additionally, we assessed the effect of anti-oxidant treatment on MC3-regulated signaling molecules namely, p21, pp38 MAPK (T180/Y182), and PARP. Pre-treatment with NAC 1 h before the addition of MC3 significantly hampered the p21 up-regulation in response to the molecule in all three cell lines. Furthermore, it attenuated the apoptotic response in HCT116 clones, as judged by a decrease in pp38 MAPK (T180/Y182) levels and cleavage of PARP (Figures 7C,D).

## Discussion

MC3 is a new gold(I) NHC complex and has previously shown remarkable anti-tumor properties against pancreatic cancer cell lines by inhibiting the catalytic activity of TrxR and the subsequent activation of the pro-apoptotic ASK1-p38 cascade (3, 4, 18). Several lines of studies have demonstrated a close relationship between p53 and the Trx/TrxR system. Trx works cooperatively with the intracellular reducing factor, REF-1 to enhance p53 DNA binding activity as well as the expression of its transcriptional target, p21 (33, 34). Furthermore, silencing *TrxR* using RNA interference has been reported to induce the ability of p53 to bind DNA (35). Noteworthy is that Trx/TrxR and REF-1 are involved in the regulation of basal p53 activity and stability but not its induction/accumulation upon DNA damage (35). This correlates well with the anti-oxidant and DNA repair functions of lower levels of p53 in non-stressed or physiologically stressed cells. However, upon severe stress p53 induces the expression of pro-oxidant genes (e.g., *PIGs, Bax*, and *Puma*), leading to apoptotic cell death (19). Considering this, it is conceivable that tumors with or without WT p53 or with mutant p53 may respond differently to TrxR inhibitors, such as MC3. The present study reveals the molecule's anti-cancer efficacy against CRC cells with distinct p53 backgrounds, however, to different degrees (Figure 7E). These effects were associated with induction of the p53 transcriptional targets, such as p21 and Bcl-2 family members as well as p38-MAPK signaling and were found to occur entirely independent of the p53 homolog, p73.

p53 represents an attractive target for anti-cancer drug development since its WT activity is disturbed in almost all human cancers either directly by mutations in the *TP53* gene or indirectly through altered expression of p53 negative and positive regulators, such as MDM2 and ARF, respectively (36). p53-deficient or -mutant tumors often acquire a more aggressive phenotype characterized by amplified chemo- and radio-resistance (8). Therefore, a lot of effort has been directed to the development of small molecules that restore the WT activity of p53, for instance PRIMA that targets mutant p53 or Nutlins which liberate WT p53 from MDM2, thereby increasing its half-life (36). Additionally, several small molecules have been reported to induce p53-related transcriptional activity in p53-null or mutant p53-harboring cells through different mechanisms that may involve p73 (30, 37, 38). Such compounds include clinically-used organometallic chemotherapeutics namely, cisplatin and oxaliplatin. With regards to gold(I) complexes, a number of studies have shown accumulation/stabilization of p53 itself as well as its anti- or pro-apoptotic mediators, such as p21, Bak, and Bax (3, 14). These findings are further supported by the work of Nandy et al. who reported an essential role for p53 in mediating the anti-tumor effects of a gold(I) NHC complex against a panel of four cancer cell lines with functional p53, including HCT116 WT (39). Besides these results, the relevance of p53 variations to the anti-cancer drug efficacy of gold(I) NHC complexes had been unclear. We here report that p53 status of HCT116 and HT-29 cells does not hamper the MC3-mediated induction of p21 and subsequent growth arrest, while it influences the pro-apoptotic responses. Importantly, MC3 is able to induce such effects at significantly lower IC_50_ values compared with similar metal-based compounds such as, auranofin and cisplatin, with having minimal toxic effects on non-cancerous cells, as previously described (18).

The tumor suppressor activity of p21 stems from its role in regulating cellular proliferation, differentiation and senescence (26). Consequently, several types of human malignancies, such as lung, cervical, and colorectal cancer have been associated with reduced p21 expression (26). Despite this, loss of function mutations in the *CDKN1A* gene, which encodes p21, are extremely rare in tumors (40), suggesting that p21 may not be a bona fide tumor suppressor, but rather exerts anti-tumor effects by synergizing with other tumor suppressor pathways (e.g., APC, ATM, and p53) or counteracting the activity of oncogenes, such as *HRAS* and *Myc* (26). We observed a profound p21 induction in all tested CRC cell lines, an effect that was accompanied by a G1-phase arrest. Interestingly, MC3-mediated growth inhibitory function was found to be significantly reduced upon p21 abrogation in HCT116 p53^−/−^ cells, but not in its WT clone. *TP53* was transcriptionally activated upon MC3 treatment in HCT116 WT cells, which at least partially accounts for the activation of its target genes, including *p21*. In p53-null or -mutant cells, however, p21 can be stimulated through pathways other than p53, such as p73, HRAS-Raf-MAPK signaling, the breast cancer susceptibility gene BRCA1, and the TGFβ/SMAD pathway (26). p73-mediated expression of p21 did not appear to be the case with MC3, since the mRNA and protein levels of its two major isoforms were strongly decreased in response to treatment. pp38 MAPK (T180/Y182) was found to be consistently up-regulated with MC3 treatment in both HCT116 clones, implicating a potential role for this signaling molecule in the observed p21 accumulation, particularly in p53-deficient cells. In contrast, no significant alteration in the level of pp38 MAPK (T180/Y182) was observed in HT-29. Therefore, MC3 may act through alternative pathways in order to activate p21 in CRC cells irrespective of the p53 status. Such pathways may include activation of the TGFβ/SMAD signaling by MC3-induced oxidative stress (data not shown), which may lead to an increase in p21 expression, as previously described (41). In support of an oxidative, p53-independent role, in mediating MC3's effect on p21 level, is attenuation of the latter's expression in response to MC3 treatment in the presence of the ROS scavenger, NAC (Figures 7C,D).

This report clearly demonstrates the ability of MC3 to trigger several pro-apoptotic molecules in WT and p53^−/−^ HCT116 cells. The disruption in cellular redox balance due to TrxR inhibition appears to be the major mechanism by which MC3 induces apoptotic cell death. In support of this, we observed increased intracellular ROS levels with treatment in the isogenic HCT116 and HT-29 cells. p53 has a direct positive role in mitochondrial biogenesis, thus its absence has been associated with reduced mitochondrial-mass and -respiration (42). This is in line with the higher intracellular ROS induction observed in the parental HCT116 cell line in response to MC3 as compared to its p53-negative counterpart. We have previously proposed that elevated ROS levels in response to MC3 relieves the inhibitory effect of reduced Trx on ASK1, leading to its activation and phosphorylation of p38 MAPK, which results in apoptosis (18). MC3 indeed led to up-regulated pp38 MAPK (T180/Y182) levels in HCT116 cells. Although, this effect was found to be substantially higher in HCT116 WT compared with HCT116 p53^−/−^, the difference did not reach statistical significance (Figure 4D). Consequently, we observed apoptotic cell death in the two clones of HCT116 cells as determined by increased annexin V-FITC binding and PARP degradation, however with higher intensity in WT p53-harboring cells. Importantly, the “p53-dependence” in MC3-mediated cytotoxic and apoptogenic response was found to happen irrespective of duration of the treatment, although toxicity was enhanced over time and with increasing doses of the molecule.

Several reports have mentioned the involvement of Bcl-2 family members and mitochondria-driven apoptosis in the anti-cancer properties of gold(I) NHC complexes (3, 4, 18, 39). On the other hand, p53 transactivation, directly and indirectly, regulates the activity of Bcl-2 family proteins (28). In this regard, the pro-survival molecule Bcl-xL, whose over-expression is associated with oncogenesis and chemo-resistance (43), was found to be consistently decreased with MC3 treatment in WT p53-harboring HCT116 cells, while no major alteration was detected in HCT116 p53^−/−^ and HT-29. In support of this is the suggested inhibitory activity of p53 on Bcl-2/Bcl-xL, which may arise from a direct protein-protein interaction that requires p53 DNA binding domain (44, 45). Therefore, tumor-derived p53 mutants, for example R273H, with impaired DNA binding ability may be also impaired for Bcl-2/Bcl-xL interaction. Western blot analyses of the pro-apoptotic member, Puma revealed a profound induction across all three cell lines, with HT-29 showing the highest responsiveness. Despite this, HT-29 cells were protected from Puma-induced apoptosis possibly through simultaneously-activated pro-survival signaling, or up-regulation of anti-apoptotic proteins, such as Bcl-2. Additionally, MC3 failed to activate p38-signaling in HT-29 at the tested conditions, which fits well with the lack of AV^+^/PI^+^ population and PARP cleavage, altogether indicating the absence of apoptosis in these cells.

An unexpected finding of the present report is a clear reduction of p73 expression in response to MC3 which was detectable across all tested CRC cell lines. p73, similar to its close homolog p53, is an important determinant of chemo-sensitivity (9). We (29) and others (30, 37) have shown that p53-targeted pathways can be restored in p53-null or -mutated CRC cells through stimulating p73 activity, as there is a substantial redundancy in the tumor suppressive functions of these two proteins (31). However, the two major isoforms of p73 (TAp73 and ΔNp73) were strongly down-regulated with MC3 treatment in the CRC cells in a dose-dependent manner. Consistent with this, we found that knocking down *TP73* in HCT116 p53^−/−^ does not rescue the MC3-mediated apoptosis as well as its anti-proliferative effects, further illustrating a p73-independent mode of action of the molecule. p73 activity/stability is controlled by an intricate network of different post-translational modifications, with a central role of the non-receptor tyrosine kinase cAbl, which initiates p73 phosphorylation (46). MAPKs including JNK and p38-signaling have been shown to mediate the cAbl-dependent phosphorylation of p73 and that their activation is sufficient to enhance p73 stability (47). Although we observed pp38 MAPK (T180/Y182) accumulation with MC3 treatment, p73 levels were found to be decreased. It is possible that components of the ubiquitin-proteasome system (UPS), for instance the E3 ubiquitin ligase ITCH or deubiquitinating enzymes (DUBs), responsible for p73 protein stability, are regulated by MC3. Consistently, we found that co-treatment with the proteasome inhibitor, MG132 counteracts the MC3-mediated degradation of p73 (data not shown). In support of the relationship between gold-containing compounds and UPS, is the work of Liu et al. (48) who reported the inhibitory effects of auranofin on two proteasome-associated DUBs namely, USP14 and UCHL5.

Altogether, our findings demonstrate the ability of MC3 to induce *in vitro* anti-cancer effects via both p53-dependent (e.g., p38 MAPK activation and apoptosis) and -independent (e.g., cell cycle inhibition) mechanisms. Given that metal-based chemotherapeutics, such as cisplatin and oxaliplatin are an essential part of the standard regimen for CRC treatment, our results may shed light on the anti-tumor potential of such compounds in the context of deficient or mutant p53–a common phenotype of the majority of human cancer types–What remains unclear however, is the alternative pathways leading to the regulation of p53 targets in CRC cells lacking WT p53 activity, as well as the mechanisms through which MC3 reduces p73 expression.

## Data Availability

All datasets generated for this study are included in the manuscript and/or the Supplementary Files.

## Author Contributions

YD and XC contributed to the design and conception of the study. JW and IO designed and synthesized the complex. YD, MA, and HC performed the experiments. YD, MA, SW, and XC analyzed and interpreted the data. YD and XC wrote the manuscript. All authors revised and approved the final version of the manuscript prior to submission.

### Conflict of Interest Statement

The authors declare that the research was conducted in the absence of any commercial or financial relationships that could be construed as a potential conflict of interest.
