# Supplementary material for: p53-Dependent Anti-Proliferative and Pro-Apoptotic Effects of a Gold(I) N-Heterocyclic Carbene (NHC) Complex in Colorectal Cancer Cells
Source: Front Oncol. 2019 May 29;9:438. doi: 10.3389/fonc.2019.00438 (PMC6558413; doi:10.3389/fonc.2019.00438)
Supplement: Supplementary file 1 [file Data_Sheet_1.docx]

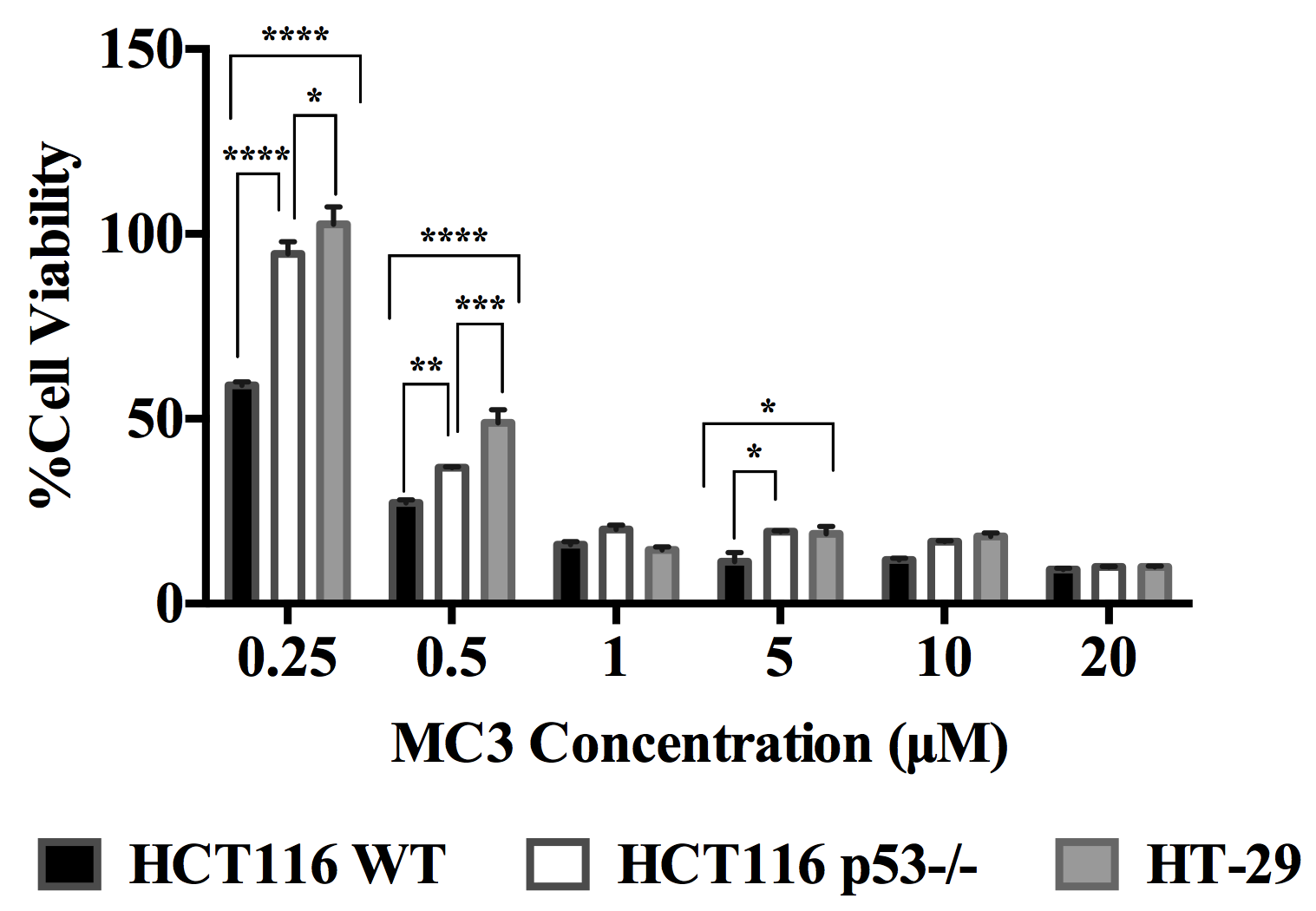


**Supplementary Figure 1.** WT p53 harboring HCT116 cells remain the most sensitive CRC cells after long-term exposure to MC3. The three CRC cells were treated with increasing concentrations of MC3 as indicated for 96 h, after which MTT assay was performed. %cell viability was calculated by normalizing to mock treatment (0.1% DMF). Multiple comparisons were employed using two-way ANOVA test and a *post-hoc* Tukey test. *, **, ***, and **** represent *p*-values less than or equal to 0.05, 0.01, 0.001, and 0.001, respectively.


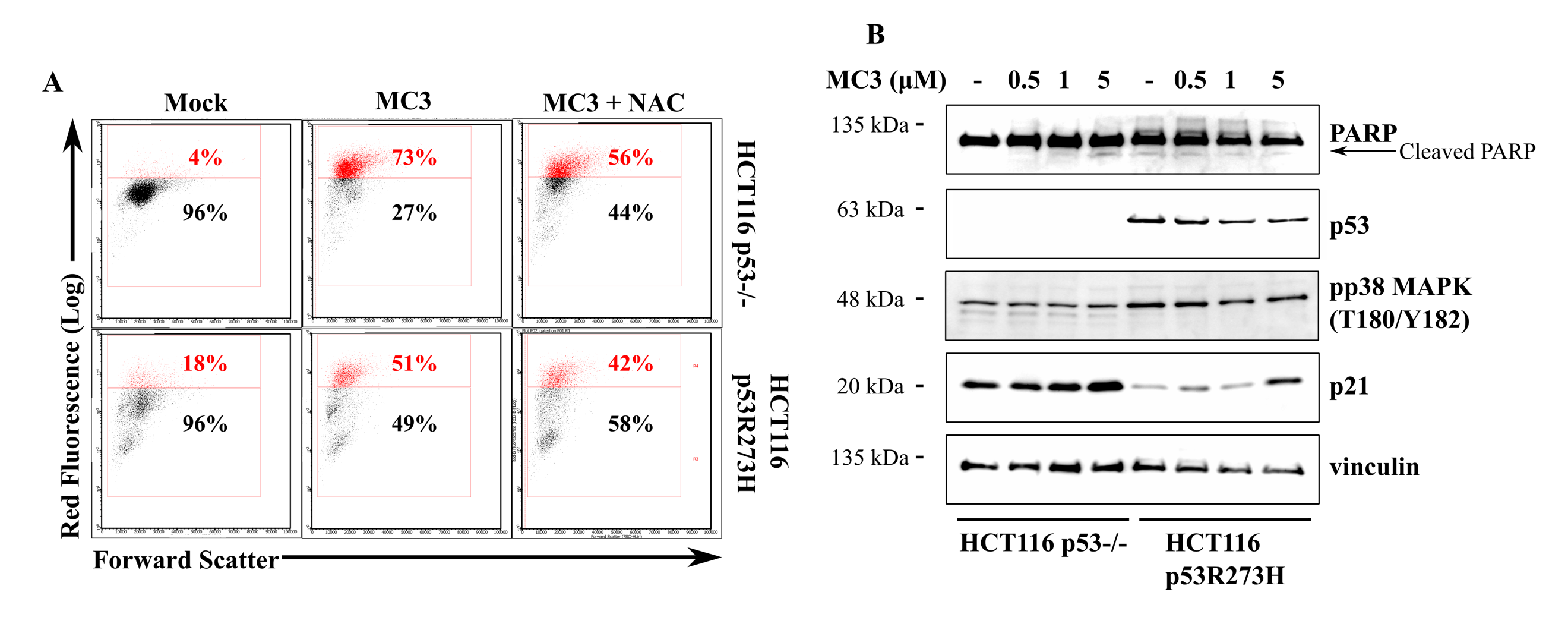


**Supplementary Figure 2.** **MC3's cellular mechanisms in mutant p53 harboring HCT116 cells.** **(A)** MC3 (5 µM, 24 h) enhances the generation of ROS in p53 R273H over-producing HCT116 cells, an effect which is compromised when cells are pre-treated with NAC (10 mM, 30 min). **(B)** HCT116 p53-/- cells over-expressing mutant p53 (R273H) demonstrate increased p21 expression, whereas they fail to induce pp38 (T180/Y182) levels and cleavage of PARP upon 24 h treatment with MC3 at the indicated concentrations, as determined by immunoblotting. 0.1% DMF served as mock treatment.


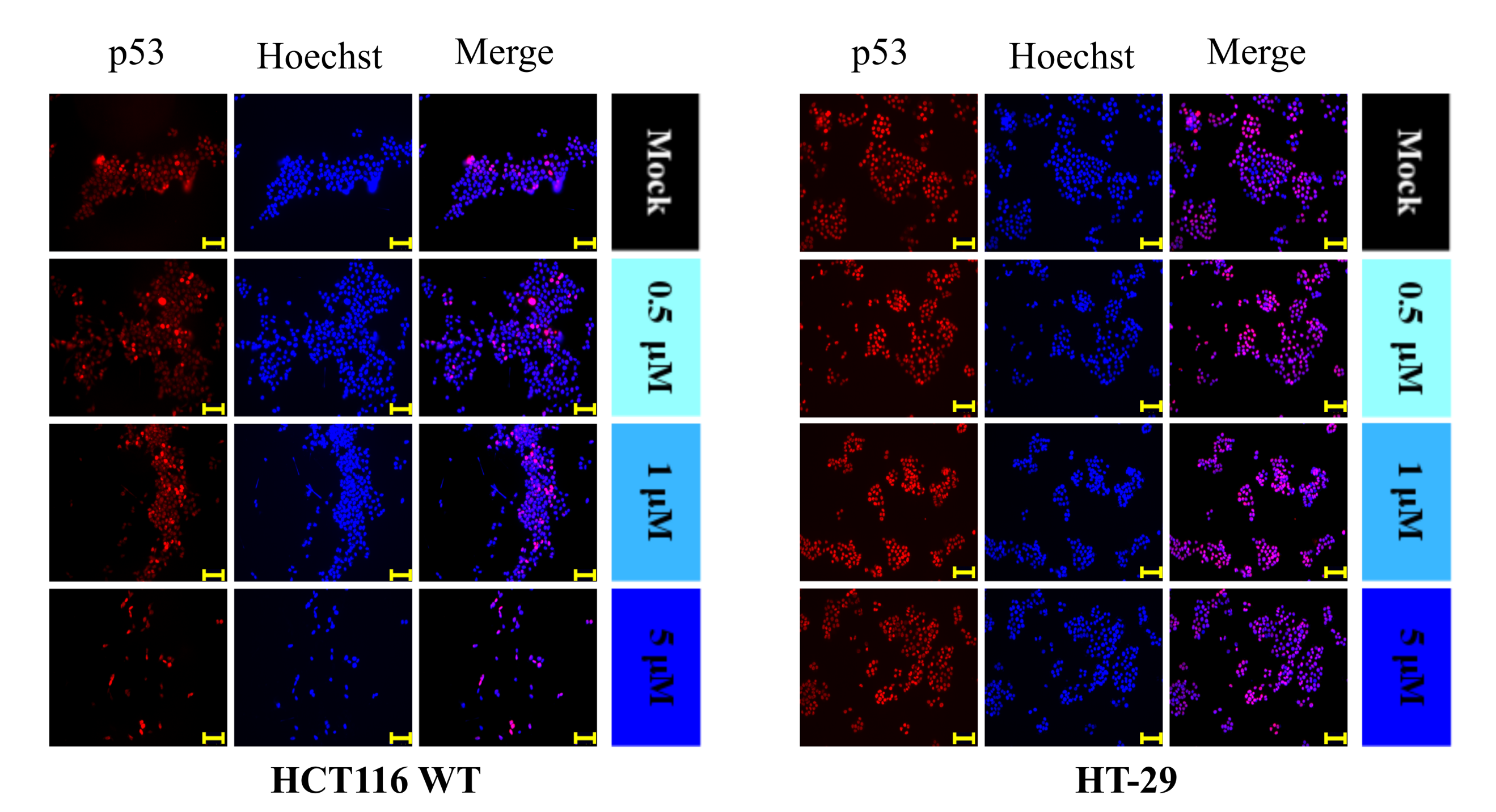


**Supplementary Figure 3.** Fluorescence micrographs of HCT116 WT and HT-29 treated with MC3 for 24 h illustrate no major change in p53 nuclear accumulation in both cell lines as compared to the respective mock (0.1% DMF) treatments. Hoechst dye was used to indicate nuclei. Scale bar: 100 µm.
